# Development and pilot testing of an interprofessional patient-centered team training programme in medical rehabilitation clinics in Germany: a process evaluation

**DOI:** 10.1186/s12909-017-0960-x

**Published:** 2017-07-14

**Authors:** Sonja Becker, Mirjam Körner, Christian Müller, Corinna Lippenberger, Manfred Rundel, Linda Zimmermann

**Affiliations:** 1 Medical Psychology and Medical Sociology, Medical Faculty, University of Freiburg, Hebelstraße 29, 79104 Freiburg, Germany; 2 Saarland University of Cooperative Education in Health Care and Welfare, Saarbrücken, Germany; 3 Celenus Kliniken GmbH, Offenburg, Germany; 4 Moving-Concept, Freiburg, Germany

**Keywords:** Interprofessional, Teamwork, Team training, Process evaluation, Chronic care, Rehabilitation

## Abstract

**Background:**

Interprofessional teamwork is considered to be a key component of patient-centred treatment in healthcare, and especially in the rehabilitation sector. To date, however, no interventions exist for improving teamwork in rehabilitation clinics in Germany. A team training programme was therefore designed that is individualised in content but standardised regarding methods and process. It is clinic specific, task related, solution focused and context oriented. The aim of the study was to implement and evaluate this training for interprofessional teams in rehabilitation clinics in Germany.

**Methods:**

The measure consists of a training of a varying number of sessions with rehabilitation teams that consists of four distinct phases. Those are undergone chronologically, each with clinic-specific contents. It was implemented between 2013 and 2014 in five rehabilitation clinics in Germany and evaluated by the participants via questionnaire (*n* = 52).

**Results:**

Staff in three clinics evaluated the programme as helpful, in particular rating moderation, discussions and communication during the training positively. Staff in the remaining two clinics rated it as not very or not helpful and mentioned long-term structural problems or a lack of need for team training as a reason for this.

**Conclusions:**

The team training is applicable and accepted by staff. It should, however, be tested in a greater sample and compared with a control group. Processes should be studied in more detail in order to determine what differentiates successful from non-successful interventions and the different requirements each of these might have.

**Electronic supplementary material:**

The online version of this article (doi:10.1186/s12909-017-0960-x) contains supplementary material, which is available to authorized users.

## Introduction

Interprofessional teamwork is regarded as a core component of patient-centred treatment in the rehabilitation sector [1–4], with studies validating its beneficial effect on organisational, staff- and patient-related outcome criteria. Organisational criteria include reduction of costs [5], minimisation of unnecessary interventions [6], and improved coordination of service [7] as well as staff binding and recruitment [6]. Staff-related outcomes affecting interprofessional teamwork are satisfaction [8], physical wellbeing [9–11], team climate [12] and efficiency [13], while patient-related criteria encompass better clinical outcomes [14, 15], acceptance of treatment [7], satisfaction [16, 17], quality of care [18] and patient safety [19, 20].

## Background

In chronic care, in particular, it is paramount that team members of different professions work together in order to reach common treatment goals. Interprofessional teams might be comprised of physicians, nurses, occupational therapists, physiotherapists, sports therapists, psychotherapists, psychologists, social workers and dieticians, depending on the chronic disease and treatment setting (e.g. community care, outpatient or inpatient rehabilitation, rehabilitation units in acute care hospitals) [21, 22]. Education in the health professions should therefore address the topic of teamwork [23–25], e.g. via team training programmes which aim to improve interprofessional teamwork [26] in order to address outputs such as team performance and patient outcomes [25]. Such team-related measures foster knowledge, abilities and attitudes of staff, and aim to boost interpersonal as well as task-related aspects in both existing and newly-formed teams.

Interventions that can be found in the literature differ in content, methods, strategies and the time they require [27], but have in common that they often consist of standardised modules with specific contents. Existing intervention studies include interprofessional training and workshops, performance feedback [28, 29] as well as the implementation of tools such as SBAR, ICF-based structure of team meetings and electronic ICF-based documentation systems [30, 31]. Positive evaluations of staff- and patient related outcomes have been found for all of these [32]: complex models that combine intervention strategies, such as workshops and tools, have for example led to reductions in length of stay [33, 34], better utilisation of capacities [34], better patient recruitment and reduced costs [28].

To date, however, despite the clear advantages, there are no interventions for interprofessional teamwork in chronic care in the German language [32]. Furthermore, interventions mainly exist in the form of standardised modules or training programs which do not necessarily fit the individual problems in rehabilitation clinics. Often there is not precisely that need in the clinics but they want to have tailored interventions to their needs. The present study is a part of the project “Development and evaluation of a concept for patient-centred team training in rehabilitation clinics (PATENT)”, which was funded by the German Federal Ministry of Education and Research (BMBF) and the German Pension Insurance. We sought to design, implement, and evaluate team training for interprofessional teams working in rehabilitation clinics in Germany in order to allow for flexible application in dependence on specific problems and to evaluate whether such an intervention is applicable and accepted by staff. We expected a very need specific, highly accepted and easily conducted intervention which could be implemented for different requests and problems in rehabilitation teams and resulted in improved teamwork.

### Development and description of the team training approach

We used a mixed methods approach, including a review of the literature [32] and a qualitative pilot study which assessed needs and topics that staff and team leaders considered important, as well as benefit factors and barriers of interprofessional teamwork [35]. Staff and team leaders in five rehabilitation clinics were surveyed via questionnaires, interviews and focus groups. Analysis of their requests revealed a broad range of topics that they would like to be assessed through team training [36]. These include the desire for better communication among the different professional groups, cooperation, leadership, patient-centredness and appreciation of each other’s work. Structural factors such as the organisation of interprofessional meetings were also mentioned frequently.

The detailed suggestions expressed in the survey were highly individual, with each clinic naming their own areas of priority. Staff and leaders differed generally in their thoughts, and there were also strong deviations apparent among the staff members and leaders in the various indication fields of the clinics. Accordingly, we designed an individualised (on the clinic level) team training programme that is standardised in methods and process, but with the content tailored to the needs of each clinic. It is additionally target-group oriented and based on actual requirements within the clinics. More specific information on the content of the programme can be found in a manual, which is also available in English language [37, 38].

The aim of the study was to implement and test the developed team training programme in medical rehabilitation practice, in order to identify changes that had been initiated through the programme.

## Methods

### Study design

The team training was implemented in five clinics in a cross-sectional study, and a process evaluation was carried out 4 weeks after the last session. A standardised questionnaire was used for evaluation. All data collection, saving, and usage of personal data was based on ethical guidelines. A positive ethical vote of the University of Freiburg is available (No 190/12).

### Description of the team training approach

Four distinct phases are undergone chronologically. The aim of the first phase is to clarify the clinic’s requests for team training and to specify the task of a selected interprofessional team together with the clinic managers. It consists of one or two meetings with our two study team trainers, the medical director and the administration manager, and possibly the leader of the team that will undergo training, if explicitly invited to attend by the clinic heads. Their request is discussed and expectations are adjusted between the two parties (trainers and clinic representatives). A request could, for example, be to “optimize the selection of patients that are discussed in the interprofessional team meeting” and to “facilitate the exchange of information regarding the patients among the different professional groups”.

The next phase consists of meetings with the trainers and actual team participating in the programme. The objective set in the first phase is then discussed with the team in order to further refine the task-oriented goal and reach a consensus with everyone concerned (staff members, team leader and management). The goal is clearly specified and conceptualised in such a way as to make it measurable and verifiable (e.g. “all the information needed to reach the rehabilitation goal is accessible for every team member”). Methods that are being used to refine the goal are e.g. the use of concept cards. As a shared goal is the heart of teamwork, this phase serves as the basis for later processes. Every team member is asked to rate in how far the goal has already been reached at this point (e.g. on a flipchart, with the help of a scale representing goal attainment).

Phase three is then introduced by asking participants to brainstorm ideas for improving realisation of the team’s common goal. These ideas are collected, prioritised and discussed with respect to their practicability and benefits, and precise steps and responsibilities are blueprinted for delivering them. Planning might include, for example, the implementation of a tool to make information accessible for all team members, or agreement on a moderator for team meetings. The process is always oriented towards resources and solutions. At the end of the training, in phase four, a procedure is outlined for stabilising results and responsibilities for the future are agreed upon.

Throughout the whole process, the trainers facilitate the process using questions known from systemic counselling.

A detailed description of the training can be found in the form of a manual [37, 38] which is published online.

### Theoretical basis

We understand coaching as an intervention which focusses on tasks, performance, processes and cooperation [39]. When planning the programme, we sought to design a clinic-specific concept, as the expectations and requests differed highly among the clinics. Therefore, we mainly focused on systemic principles that are open, solution-focused and resource-oriented [40], in order to give trainers more of an heuristic advice on how to support teams in the process of reaching goal attainment. Based on the approach by Hackman and Wageman [41], it intents to “help members make co-ordinated and task-appropriate use of their collective resources in accomplishing the team’s work”. It is therefore consultative in nature and focusses on tasks and task performance, not on interpersonal relationships.

The training combines different approaches [42]: While the systemic approach serves as a basis, it is complemented by the task-oriented and the process- or solution-focused approach [43]. Therefore, the focus lies on individual needs of staff members in each clinic and all team members are included in the problem-solving process. This way, competencies of all members are being used and staff’s identification with the solutions is highest. At the same time, the team remains autonomous and holds the competencies for problem solution. The trainer supports team members in finding their own goals, identifying problems in cooperation that hinder goal achievement and in developing solutions via goal-directed behavior [43, 44].

The trainers were mainly members of the research team Medical Psychology and Medical Sociology of the Medical Faculty, University of Freiburg, except for one external trainer. Trainers were part of the study team and had been part of the programme’s design. All were trained and experienced in systemic coaching. They helped the team formulating and working on their goal by using techniques and questions from systemic counseling which aim to help clients make use of their own resources. They structured and moderated the meetings by summarising results of former meetings, having at hand methods to enable group discussions and creative processes and guiding the process through the four introduced phases. They were included in the development of the training and were therefore especially skilled to carry out the training. Each session was guided by two trainers.

The intervention is highly innovative, as, to our knowledge, there are no team training interventions in rehabilitation that consist of a standardised process which can be carried out in order to solve individual problems within a clinic. The innovation of the intervention consists of the combination of different team development approaches and the possibility to give trainers an action guideline and a toolkit [45] which helps them foster individual change processes within a team.

### Implementation of team training and data collection

The training was conducted in the five clinics during 2013 and 2014. Initially, 114 clinics of different indications in medical rehabilitation in the study area were addressed, of which 24 demonstrated interest in taking part in the study. In the end, 10 clinics remained to undergo the whole process, of which 5 were randomly assigned to the control−/intervention group by matching the clinics according to size and indication field and then blindly assigning clinics to the intervention group (and the matching one to the control group, respectively). Only the intervention group received the training and filled out the questionnaire on process evaluation, whereas the control group received no intervention. In each clinic, two trainers were responsible for the sessions. The number of sessions and timespan between these differed between clinics, ranging from one to six within the timespan of one to 15 months (see Table 1). This variability is due to the needs- specific approach, which takes into account the individual nature of processes and the time they take in the different clinics.

**Table 1:** Overview of clinics and sessions

Clinic	No. of sessions	Intervention period
1	3	07/13–02/14
2	6	10/13–06/14
3	4	11/13–05/14
4	3	03/14–07/14
5	1	09/13

### Questionnaire

The questionnaire contained the adapted German instrument “Training measures and their success” [46], which contains standardised items that have to be answered on a 10- point Likert scale ranging from 0 to 100%. For calculation of results, they are recoded ranging from 0 to 10. The second part of the questionnaire contained items that have been set up by the research team and have to be answered on a Likert scale ranging from 1 to 4. Additionally, participants had the possibility to give a free response on what they appreciated or criticized about the training and which improvements they suggested. The items of the questionnaire can be summed up in the following five scales, which were built thematically and validated via factor analysis by the authors (Cronbach’s α see Table 3):

A. Satisfaction with the team training (Item 1–5)
B. Personal development through the team training (Item 6–9)
C. Effects of the team training (Item 10–15)
D. Interdisciplinary through the team training (Item 16–21)
E. Sustainability of the team training effects (Item 22)

Items 1 to 15 are to be answered on a 0 (not true at all) to 100 (totally true) Likert scale, items 16 to 22 are to be answered on a 1 (not true at all) to 4 (totally true) Likert scale. A list of items and scales, respectively, is included as additional files (Additional file 1: List of items).

Additionally, the questionnaire contained the two open questions “What did you like especially about the team training?” and “What did you dislike?”, which were indicated with a happy and a sad face, respectively. There were several spare lines on the bottom of the questionnaire which participants could use in order to leave any kind of comment on the training.

### Data analysis

Data was analysed using IBM SPSS Statistics 21. As the training program is highly individual, MANOVAs were carried out in order to investigate potential differences between the clinics. Post hoc analyses were then carried out to differentiate more and less successful clinics, which gives information on the success of the training. Optional comments at the end of the questionnaire are considered in order to help understand potential differences between the clinics.

## Results

### Sample

The sample consisted of five teams made up of *n* = 52 professionals in total. Table 2 presents the distribution of professions among the different clinics. Twenty five of the participants were female, 17 male and the rest did not give information on their gender. The majority of staff members were aged 50–59 (36.5%), followed by those aged 40–49 (19.2%), 30–39 (13.5%), and younger than 30/older than 59 (7.7% respectively), with 15.4% missing answers. More than 50% had been working in their clinic for more than 8 years, and 17% held a leading position within their team. The number of training sessions in each clinic was determined according to the individual training content, and ranged from 1 to 6. Overall, 3% took part in only one session, 25% of staff took part in two sessions, 17% in three, 15% in four sessions and 6% in six sessions, with 34% missing answers. Not all staff took part in every session that was offered due to illness or other reasons for absence at the time of training.

**Table 2:** Constitution of interprofessional teams in clinics 1 to 5

Profession	Clinic 1	Clinic 2	Clinic 3	Clinic 4	Clinic 5	Total
Physician	8	2	2	1	0	13
Nurse	1	4	2	0	0	7
Physiotherapist	2	4	0	1	2	9
Psychologist	1	1	2	1	1	6
Speech Therapist	0	2	0	0	0	2
Social Worker	0	0	2	0	0	2
Dietician	0	0	0	1	0	1
Occupational Therapist	0	0	0	0	1	1
Other/missing	1	0	3	5	2	11
*Total n*	13	13	11	9	6	52

### Descriptive statistics

Overall, clinics 1, 2 and 4 demonstrated positive to highly positive evaluation results for the training. Means of the item “All in all, I liked the team training” (answers from 1 to 10) were M = 7.4 (SD = 2.8) in clinic 1, M = 7.7 (SD = 2.5) in clinic 2 and M = 7.4 (SD = 2.1) in clinic 4. Feedback in clinics 3 and 5 was less favourable, however, with the former rating this item M = 2.5 (SD = 2) and the latter M = 3.3 (SD = 2.9). Means for the item “I would recommend the team training to others” were M = 7.8 (SD = 3.2) in clinic 1, M = 7.7 (SD = 2.3) in clinic 2 and M = 7.3 (SD = 1.7) in clinic 4. Again, staff of clinics 3 and 5 rated this item rather negatively, with M = 2.5 (SD = 2.3) in clinic 3 and M = 3.8 (SD = 3.7) in clinic 5. On the level of the 5 subscales results are similar, with clinics 1, 2 and 4 demonstrating a tendency to more affirmative and clinics 3 and 5 more negative evaluations of the training and its effects. Table 3 presents means and standard deviations for the five subscales for each clinic as well as Cronbach’s α for scales A-D (as E refers to one item only).

**Table 3:** Means and standard deviations on the scales A to E presented separately for each clinic

Clinic	Scale A	Scale B	Scale C	Scale D	Scale E
1	8.4 (1.8)	6.2 (2.9)	6.6 (2.3)	3.1 (.6)	3.1 (1.0)
2	7.7 (2.1)	6.0 (2.5)	5.0 (2.0)	2.6 (.4)	2.7 (.5)
3	2.9 (1.7)	2.2 (1.5)	1.0 (1.7)	2.0 (.4)	2.0 (.5)
4	7.0 (1.8)	4.5 (3.1)	4.9 (3.2)	2.5 (.5)	2.4 (.5)
5	3.6 (2.9)	2.9 (2.6)	1.6 (2.1)	2.2 (.7)	1.7 (.8)
Cronbach’s α	.97	.79	.91	.86	-

*A* Satisfaction with the team training (Range 1–10), *B* Personal development through the team training (Range 1–10), *C* Effects of the team training (Range 1–10), *D* Interdisciplinarity through the team training (Range 1–4), *E* Sustainability of the team training effects (Range 1–4)

### Differences between the clinics

The clinics differ in their evaluation of the team training on a scale level. Staff in clinics 1, 2 and 4 view it overall more favourably than staff in clinics 3 and 5. They also evaluate it positively in absolute terms, demonstrating means higher than 5.0 on the scales satisfaction, personal development and effects of the team training (A-C), and higher than 2 for interdisciplinarity (D) and sustainability (E), whereas the staff of clinics 3 and 5 evaluate it as not very helpful over all scales.

In a subsequent step, we calculated a MANOVA in order to test for significant differences on a scale level between the clinics. The difference between the clinics is statistically significant for all scales (*p* < .05), demonstrating high effect sizes (see Table 4).

**Table 4:** Comparisons of means among the clinics for scales A to E

	Range 0–10			Range 1–4	
	Scale A	Scale B	Scale C	Scale D	Scale E
F (df1, df2)	8.7 (4,4)	4.6 (4,4)	8.6 (4,4)	5.1 (4,.4)	3.9 (4,4)
Significance	<.001	<.01	<.001	<.01	<.05
Partial Eta^2	0.52	0.37	0.52	0.39	0.33

As the results showed that clinics differed in their evaluation of the team training, post hoc contrasts were calculated in order to analyse which clinics differ on which specific scales in detail.

The post hoc contrasts (Scheffé) reveal that some differences between clinics 1 and 2 and clinics 3 and 5, respectively, are statistically significant. Clinic 1 demonstrates higher values on all scales apart from scale E (sustainability) compared to clinic 3 (*p* < .05), and on scale C (effects of the team training) compared to clinic 5 (*p* < .05). The training is judged significantly better on scales A to C in clinic 2 than in clinic 3 (*p* < .05), but not better than in clinic 5. Clinic 4, on the other hand, displays no significant differences to any of the other clinics. (Additional file 2: Table S5) sums up the results of the post hoc tests.

### Free comments

Looking at the optional comments that staff made in the questionnaire, it is possible to form hypotheses on which aspects of the team training were more or less helpful and why it was rated significantly better in some clinics than in others. Out of the 71 participants, 52 made optional comments.

In the following, comments shall be presented in order to shed light on the differences between the clinics that were found in the statistical analyses. For this purpose, comments of staff that rated the training as little successful are compared with comments of staff that rated the training as successful. Staff in clinic 3 wrote that interpersonal conflicts which had been present but neglected beforehand became apparent through and during the training. However, it was not possible due to the structure and objectives of the training to solve those conflicts (e.g. one participant wrote “Familiar problems were brought up again without finding satisfying solutions, with basic conditions that are hard to change.”). This possibly led to the unfavourable evaluation in clinic 3. In clinic 5, however, the lack of perceived development by staff can also be explained by the comments, which included that after only one session staff decided that teamwork was already successful and they did not need further training. As a result, one professional wrote that s/he rated the items on developments due to the intervention as “negative”, since s/he did not feel that circumstances prior to it had been bad: “Team meetings and information processing between the groups had already been very good before the training” (participant of clinic 5). This would explain why the staff here did not perceive any positive development.

In clinics 1, 2 and 4 the comments, like the rating of the items, reflect a more positive evaluation. As to the things they liked, staff mentioned “the good moderation and resulting discussion in the team that in the end led to a development” (clinic 1), the “claim for self-development” (clinic 1) and the “honest and open discussion” (clinic 2). One participant in clinic 4 stated that s/he found the training to be especially “constructive”. Moderation was commented on favourably by several participants, with one professional (clinic 2) saying that it served as a “role model” for interprofessional communication. Further affirmative remarks related to individual methods such as summaries and minutes of the sessions, as they helped staff to recall the discussions after the sessions.

## Discussion

The training to improve interprofessional teamwork was tested in five rehabilitation clinics in south-west Germany in order to identify changes that had been initiated through the training. The evaluation results are heterogeneous for the different clinics. Staff in three of the clinics commented favourably, rating the moderation, in particular, as positive and noting that reflecting together on team processes during the training could serve as a basis for improved communication during team meetings. Staff in the remaining two clinics, who rated the training as not very or not helpful, argued a) that awareness of long-standing structural problems was regenerated within the team, which led to conflicts that could not be solved within the scope of this short intervention, or b) that teamwork had already been good before the intervention and staff consequently saw no need for team training. Those results substantiate the hypothesis that the programme designed and used in this survey can help interprofessional teams in chronic care to improve their communication and reach their treatment goals. They also show, however, due to their heterogeneity, that it is imperative when implementing team training to analyse the status quo and the needs and expectations of the contractor, i.e. usually the head of the clinic, and the team. This corresponds with suggestions in the literature [47] that point out that team training in the health care context should ensure the relevancy as well as the careful design of the measure. One of the most central parts of successful team training, as stated earlier, is to have a clear and common goal that contractor and counsellor share. This clarification process is part of our team training programme, but, as the results show, has not always been achieved satisfactorily.

The results also confirm that it is possible to implement team training for rehabilitation clinics in Germany that is adjustable in terms of content but standardised in its process. The process of finding and agreeing upon a common goal for all professions and improving the processes within the team with regard to that goal was mostly successful. This underpins the idea that a common goal is the crucial element in a team [47] and the process of defining goals, setting deadlines and following them in order to reach those goals is considered a vital component of participative team interventions [25, 48]. The important role of working on specific tasks, as is done in our solution-focussed intervention, is also supported by models on healthcare team effectiveness using the input-process-output model of teamwork [14].

Although there is a high variability in results among the clinics, we think that our findings can help both to improve future research and practice. While objective data on the impact in terms of a pre-post evaluation of the intervention is discussed elsewhere [49, 50], the process evaluation presented here shows that the process of change in team interventions should be studied in order to evaluate in more detail those processes that differed between the successful and less successful clinics, i.e. clinics where staff rated the training as helpful/not helpful in improving goal attainment. This is analogous to research in psychotherapy, where the focus on the process tells us more about the mechanisms of change [51]. As such studies on psychotherapy change processes show, interventions usually broaden the range of variance in problem severity. It follows, then, that in coaching an intervention such as team training is quite probably able to both boost and impair goal attainment, as was shown in clinics 3 and 5 vs 1, 2 and 4. Adverse effects of coaching occur rather frequently and in a broad range of dimensions, such as factors associated with clients, the coach, their relationship or context and structures [51]. Apart from those possible failures due to unsuccessful implementation, underlying assumptions and theories of the intervention can be wrong [48, 52]. The data on individual processes that have taken place in the clinics can therefore help in understanding which factors lead to adverse effects and what precautions should be taken in future implementations.

The data of our study, however, suggests that in one clinic the structures did not support the team training and in another clinic staff saw no need for it. This underlines that counsellors should take into account the individual problems and status quos of contractors and decide whether team training such as ours, which is based mainly on resources and problem solutions, is able to further teamwork in a specific clinic or whether supervision or other measures that focus more on structural aspects are needed. This would correlate with findings from a study where a staff intervention supposedly failed in parts because it did not focus enough on the role structures, such as of the unit and department leaders [48]. It has to be noted, however, that due to the nature of the study it was not the clinics who asked for help, but the research team itself that offered team training as part of a study if the clinics were interested. Also, pre-intervention working conditions have been proven relevant for staff participation in the implementation of interventions [53]. From a systemic point of view, it seems reasonable that change induced in a subsystem can lead to dissonance when the context does not change or is not ready for change: hence, basic conditions within the clinic are equally decisive for successfully implementing long-term change in health care, and contractors should take into account in how far they are willing to give way to change.

Hand in hand with this goes the aspect of staff participation. It has been stated that staff participation in terms of joint planning and delivery of a teamwork intervention is crucial for its successful implementation [53]. Although team members and leaders were included in our initial design phase, the open nature of the process would perhaps have needed a closer focus on staff participation throughout the entire implementation period. It is possible that, due to differing expectations among staff and leaders, the intervention could not be as successful in clinic 3 as in the other clinics. This draws our attention to change management research, which identifies change participation as an important mediator between organisational and supervisor support and positive change evaluation [54].

Overall, qualitative methods could possibly help us create insight into why the quantitative results turn out as they do, and in turn enable us to get a better picture of processes that lead to relative success or failure of an intervention [48, 55]. Future studies should therefore generate such in-depth information in order to help give educators more information on processes that they should especially focus on in the clinics because they are related to crucial changes. Additionally, an analysis of pre-intervention status quo in the clinics could help to investigate whether certain resources that can be found in the successful clinics facilitate the implementation of a team intervention [53]. Effects of team training on measures of patient satisfaction, patient outcomes and clinic efficiency should be taken into account in future studies to investigate outcomes on an organizational level.

### Limitations

Because of the rather small sample size the data has to be viewed with caution. The results of the inferential statistics, in particular, can only be seen as pointers for a better understanding of the different processes among the clinics. The intervention should be tested in a bigger sample and compared with control clinics, allowing analyses that compare different indication fields. The variability of staff who took part in the training and the variable number of sessions the different clinics received can be seen as a limitation. However, this is part of the needs- specific approach. Moreover, not all staff members who had taken part in at least one session filled out the questionnaire. Response rates ranged from 38 to 69%, with the lowest in clinic 5, meaning that selection bias is possible. Rather low response rates like this are often seen in organizational contexts [56] and are probably among other factors due to high workload of staff. It should, however, be mentioned that the clinic with the lowest response rate demonstrated rather negative evaluations.

Finally, the results show effects for a short time frame (the survey questionnaire was distributed about 4 weeks after the end of the training). A follow-up inquiry should therefore be carried out a few months after the last session in order to evaluate long-term effectiveness. The information regarding why the training was evaluated more negatively in two clinics is based on the free-text responses in the questionnaire.

## Conclusions

All in all, first results confirm that the developed team training programme is accepted by staff and is suitable for implementation in rehabilitation clinics. Those participants who evaluated its effects positively would recommend it and believe that it will have long-lasting effects in their team. For the future, it should be tested in a cluster-randomised controlled study and a greater sample. Processes of implementation should also be studied in more depth in order to find out what differentiates successful from non-successful clinics in terms of requirements and process variables.

## Additional files

Additional file 1: List of items (translated from German). List of items used in the questionnaire, translated from German by the authors. (DOCX 15 kb)

Additional file 2: Table S5. Results of post hoc tests for scales A to E and clinics 1 to 5. (DOCX 18 kb)
