# Supplementary material for: Development and pilot testing of an interprofessional patient-centered team training programme in medical rehabilitation clinics in Germany: a process evaluation
Source: BMC Med Educ. 2017 Jul 14;17:120. doi: 10.1186/s12909-017-0960-x (PMC5512750; doi:10.1186/s12909-017-0960-x)
Supplement: Supplementary file 1 — List of items (translated from German). List of items used in the questionnaire, translated from German by the authors. (DOCX 15 kb) [file 12909_2017_960_MOESM1_ESM.docx]

## List of items (translated from German)

**Item Scale**

| 1. All in all, I liked the team training. |  |
| --- | --- |
| 1. I would recommend the team training. |  |
| 1. The moderation of the training was good. |  |
| 1. The moderators provided adequate time for interprofessional exchange. |  |
| 1. Participation in the team training was especially useful for our work in the team. |  |
| 1. Through the team training my attitude towards interprofessional teamwork changed in a positive way. |  |
| 1. Through the team training the task of our team became clearer to me. |  |
| 1. The content of the team training was relevant for my everyday clinical praxis. |  |
| 1. Since the team training I have been more content with my work. |  |
| 1. Since the team training our team has been working more effectively over all. |  |
| 1. Through the team training workflows could be simplified. |  |
| 1. Through the team training the climate within our team has improved. |  |
| 1. Since the team training all professional groups are involved more strongly in the team meeting. |  |
| 1. Through the team training, I feel I can satisfy the professional requirements better. |  |
| 1. Through the team training we have reached our goal. |  |
| 1. Since the team training our team meetings have been more effective. Content, goals and process are clear. |  |
| 1. The time available for team meetings is being used in an optimal way. |  |
| 1. Since the team training, there are clearer rules for information exchange. |  |
| 1. Since the team training I get important impulses for my work during the team meetings. |  |
| 1. Since the team training my task in the team has become clearer. |  |
| 1. Adressing the interdisciplinary team meeting in the team training has had a positive effect on patient treatment coordination. |  |
| 1. The team training will have sustainable effects on teamwork in our clinic. |  |
