# Supplementary material for: Development and pilot testing of an interprofessional patient-centered team training programme in medical rehabilitation clinics in Germany: a process evaluation
Source: BMC Med Educ. 2017 Jul 14;17:120. doi: 10.1186/s12909-017-0960-x (PMC5512750; doi:10.1186/s12909-017-0960-x)
Supplement: Supplementary file 2 — Results of post hoc tests for scales A to E and clinics 1 to 5. (DOCX 18 kb) [file 12909_2017_960_MOESM2_ESM.docx]

| Table 5 | | | | | | | | | | | | | | | | | | | | | | | | | | | | | | | | |  |  |
| --- | --- | --- | --- | --- | --- | --- | --- | --- | --- | --- | --- | --- | --- | --- | --- | --- | --- | --- | --- | --- | --- | --- | --- | --- | --- | --- | --- | --- | --- | --- | --- | --- | --- | --- |
| *Results of post hoc tests for scales A to E and clinics 1 to 5* | | | | | | | | | | | | | | | | | | | | | | | | | | | | | | | | |  |  |
|  |  |  | Scale A | | | | |  | Scale B | | | | |  | Scale C | | | | |  | Scale D | | | | |  | Scale E | | | | |  | | |
|  |  |  | Clinic | | | | |  | Clinic | | | | |  | Clinic | | | | |  | Clinic | | | | |  | Clinic | | | | |  | | |
|  |  |  | 1 | 2 | 3 | 4 | 5 |  | 1 | 2 | 3 | 4 | 5 |  | 1 | 2 | 3 | 4 | 5 |  | 1 | 2 | 3 | 4 | 5 |  | 1 | 2 | 3 | 4 | 5 |  | | |
| Scale A | Clinic | 1 |  |  |  |  |  |  |  |  |  |  |  |  |  |  |  |  |  |  |  |  |  |  |  |  |  |  |  |  |  |  | | |
|  |  | 2 | 0.4 |  |  |  |  |  |  |  |  |  |  |  |  |  |  |  |  |  |  |  |  |  |  |  |  |  |  |  |  |  | | |
|  |  | 3 | 5.2* | 4.8* |  |  |  |  |  |  |  |  |  |  |  |  |  |  |  |  |  |  |  |  |  |  |  |  |  |  |  |  | | |
|  |  | 4 | 1.4 | .9 | 3.8 |  |  |  |  |  |  |  |  |  |  |  |  |  |  |  |  |  |  |  |  |  |  |  |  |  |  |  | | |
|  |  | 5 | 3.8 | 3.4 | 1.4 | 2.4 |  |  |  |  |  |  |  |  |  |  |  |  |  |  |  |  |  |  |  |  |  |  |  |  |  |  | | |
|  |  |  |  |  |  |  |  |  |  |  |  |  |  |  |  |  |  |  |  |  |  |  |  |  |  |  |  |  |  |  |  |  | | |
| Scale B | Clinic | 1 |  |  |  |  |  |  |  |  |  |  |  |  |  |  |  |  |  |  |  |  |  |  |  |  |  |  |  |  |  |  | | |
|  |  | 2 |  |  |  |  |  |  | 0.7 |  |  |  |  |  |  |  |  |  |  |  |  |  |  |  |  |  |  |  |  |  |  |  | | |
|  |  | 3 |  |  |  |  |  |  | 4.5* | 3.8* |  |  |  |  |  |  |  |  |  |  |  |  |  |  |  |  |  |  |  |  |  |  | | |
|  |  | 4 |  |  |  |  |  |  | 2.1 | 1.4 | 2.4 |  |  |  |  |  |  |  |  |  |  |  |  |  |  |  |  |  |  |  |  |  | | |
|  |  | 5 |  |  |  |  |  |  | 3.4 | 2.8 | 1.0 | 1.4 |  |  |  |  |  |  |  |  |  |  |  |  |  |  |  |  |  |  |  |  | | |
|  |  |  |  |  |  |  |  |  |  |  |  |  |  |  |  |  |  |  |  |  |  |  |  |  |  |  |  |  |  |  |  |  | | |
| Scale C | Clinic | 1 |  |  |  |  |  |  |  |  |  |  |  |  |  |  |  |  |  |  |  |  |  |  |  |  |  |  |  |  |  |  | | |
|  |  | 2 |  |  |  |  |  |  |  |  |  |  |  |  | 1.7 |  |  |  |  |  |  |  |  |  |  |  |  |  |  |  |  |  | | |
|  |  | 3 |  |  |  |  |  |  |  |  |  |  |  |  | 5.9* | 4.2* |  |  |  |  |  |  |  |  |  |  |  |  |  |  |  |  | | |
|  |  | 4 |  |  |  |  |  |  |  |  |  |  |  |  | 2.3 | 0.6 | 3.6 |  |  |  |  |  |  |  |  |  |  |  |  |  |  |  | | |
|  |  | 5 |  |  |  |  |  |  |  |  |  |  |  |  | 5.0* | 3.3 | 0.9 | 2.7 |  |  |  |  |  |  |  |  |  |  |  |  |  |  | | |
|  |  |  |  |  |  |  |  |  |  |  |  |  |  |  |  |  |  |  |  |  |  |  |  |  |  |  |  |  |  |  |  |  | | |
| Scale D | Clinic | 1 |  |  |  |  |  |  |  |  |  |  |  |  |  |  |  |  |  |  |  |  |  |  |  |  |  |  |  |  |  |  | | |
|  |  | 2 |  |  |  |  |  |  |  |  |  |  |  |  |  |  |  |  |  |  | 0.5 |  |  |  |  |  |  |  |  |  |  |  | | |
|  |  | 3 |  |  |  |  |  |  |  |  |  |  |  |  |  |  |  |  |  |  | 1.1* | 0.6 |  |  |  |  |  |  |  |  |  |  | | |
|  |  | 4 |  |  |  |  |  |  |  |  |  |  |  |  |  |  |  |  |  |  | 0.4 | 0.1 | 0.7 |  |  |  |  |  |  |  |  |  | | |
|  |  | 5 |  |  |  |  |  |  |  |  |  |  |  |  |  |  |  |  |  |  | 0.9 | 0.4 | 0.2 | 0.5 |  |  |  |  |  |  |  |  | | |
|  |  |  |  |  |  |  |  |  |  |  |  |  |  |  |  |  |  |  |  |  |  |  |  |  |  |  |  |  |  |  |  |  | | |
| Scale E | Clinic | 1 |  |  |  |  |  |  |  |  |  |  |  |  |  |  |  |  |  |  |  |  |  |  |  |  |  |  |  |  |  |  | | |
|  |  | 2 |  |  |  |  |  |  |  |  |  |  |  |  |  |  |  |  |  |  |  |  |  |  |  |  | 0.4 |  |  |  |  |  | | |
|  |  | 3 |  |  |  |  |  |  |  |  |  |  |  |  |  |  |  |  |  |  |  |  |  |  |  |  | 1.1 | 0.7 |  |  |  |  | | |
|  |  | 4 |  |  |  |  |  |  |  |  |  |  |  |  |  |  |  |  |  |  |  |  |  |  |  |  | 0.5 | 0.1 | 0.6 |  |  |  | | |
|  |  | 5 |  |  |  |  |  |  |  |  |  |  |  |  |  |  |  |  |  |  |  |  |  |  |  |  | 1.3 | 0.9 | 0.2 | 0.8 |  |  | | |
|  |  |  |  |  |  |  |  |  |  |  |  |  |  |  |  |  |  |  |  |  |  |  |  |  |  |  |  |  |  |  |  |  | |  |
| *Note.* * Mean difference is significant on a .05 level | | | | | | | | | | | | | | | | | | | | | | | | | | | | | | | | |  |  |
